# Language Assessment Literacy of Teachers

**DOI:** 10.3389/fpsyg.2022.864582

**Published:** 2022-05-05

**Authors:** Fanrong Weng, Bin Shen

**Affiliations:** ^1^Department of English, University of Macau, Taipa, Macao SAR, China; ^2^School of Foreign Languages, Fuzhou University, Fuzhou, China; ^3^Center for Foreign Language Education and Teaching, School of Foreign Languages, Fuzhou University, Fuzhou, China

**Keywords:** language assessment literacy, language teachers, teacher education, training needs, professional development

## Abstract

Language assessment literacy (LAL) is a significant component of language teachers’ expertise but is also a challenging task for most language teachers. To date, there have been relatively few studies examining the research on teachers’ LAL during these decades. To fill this void, this article reviews the conceptualizations of LAL and relevant empirical studies published from 1991 to 2021. It first analyzes various conceptualizations of LAL. Then in examining the empirical studies on teachers’ LAL, five major themes emerge: (i) teachers’ LAL levels; (ii) factors influencing LAL; (iii) language teachers’ assessment training needs; (iv) language assessment training courses; and (v) LAL development through reflection. Finally, future research directions on teachers’ LAL are discussed.

## Introduction

Language assessment literacy (LAL) plays an increasingly important role in language education and constitutes an integral part of language teachers’ professional competence (Popham, 2009; Kremmel and Harding, 2020). The teachers who are language assessment literate can design and administer effective testing activities, interpret students’ scores accurately, formulate appropriate teaching plans and make rational education decisions. However, teachers’ insufficient LAL may lead to poorly designed language assessments, incorrect interpretation of test results and irrational educational decisions, all of which may have negative consequences for students.

However, despite its crucial role in language teaching and assessment, teachers’ LAL remained inadequate (Berry et al., 2017; Xu and Brown, 2017). TESOL programs for pre-service teachers incorporate insufficient language assessment contents (Jeong, 2013), and limited language assessment training opportunities are provided to in-service teachers (Crusan et al., 2016). Additionally, education policymakers and university or school administrators were at fault for not ensuring teachers are well trained before starting their teaching careers (Coombe et al., 2012). These factors together inhibit teachers’ LAL development.

Recognizing the importance of language teachers’ LAL and the needs for teacher development, a number of studies have been performed to conceptualize LAL, investigating teachers’ LAL and supporting resources in different contexts and from different perspectives. However, relevant research is “still in its infancy” (Fulcher, 2012, p. 117), and more research on this topic is needed to advance LAL scholarship. To provide a comprehensive picture of current research and lay the foundation for future LAL studies, this review will first examine the conceptualizations of LAL and the empirical studies on teachers’ LAL, then provide implications and future directions.

## Selection of Studies

This article reviews the studies on LAL from 1991 to 2021, including journal articles, books, book chapters, and conference proceedings. The literature search was divided into two stages. During the first stage, searches were performed in Web of Science, Google Scholar, Educational Resource Information Center (ERIC), and Scopus using a combination of the following keywords: “language assessment literacy” “teacher assessment literacy” “language teacher education,” and “teacher professional development.” Additionally, the time “since 1991” was chosen, since the concept of assessment literacy was first proposed in this year by Stiggins (1991). The articles retrieved were screened according to the following criteria: (i) studies aimed at language teachers; and (ii) studies discussing language assessment literacy. And the studies that met the criteria were retained. In the second stage, the citations of these studies were examined in order to identify potential new sources. The newly retrieved literature was then screened using the same criteria.

The selected studies were analyzed using a content analysis approach and were classified according to their themes. The analyses of the studies will be presented in the sections that follow.

## An Overview of LAL Research From 1991 to 2021

This review covers three decades of LAL research. During the first decade (1991–2000), the concept of assessment literacy firstly emerged in the literature of educational measurement. Nevertheless, it was not until the early 2000s that assessment literacy was extensively examined by the language assessment community.

In the decade from 2001 to 2010, within the umbrella term of assessment literacy, LAL was first proposed. The research during this period primarily focused on conceptualizing LAL and several LAL frameworks with an emphasis on language teachers were proposed. In addition, two crucial views arose from the relevant research. First, LAL should be considered separately from assessment literacy in general education, due to the complexities inherent in testing and assessing language abilities and communicative competence (Harding and Kremmel, 2016). Second, since language assessment is closely linked to students’ lives, educational policies and society, academics argued that LAL research should be reframed from a broader social constructivism lens, reflecting current needs of society (Inbar-Lourie, 2016), rather than confined in the applied psychometrics-based testing culture (Wu, 2018).

The decade that followed (2011–2021) saw the continued development of LAL research. During this period, additional efforts were made to conceptualize LAL in a variety of contexts, and empirical studies on teachers’ LAL arose and flourished. During this period, LAL was gradually conceptualized as a multidimensional and developmental concept. In addition, as a continuation of the previous phase, research on LAL has continued to emphasize the significance and influence of social contexts. LAL was considered as a highly contextualized concept and language teachers in different social contexts required distinct LAL profiles. Furthermore, the growing interest in LAL has led to a plethora of empirical studies examining teachers’ LAL levels, language assessment training needs, and professional development programs.

## Conceptualizations of LAL

As mentioned above, the concept of assessment literacy was first proposed by Stiggins (1991), who defined assessment literates as the stakeholders who “have a basic understanding of the meaning of high- and low-quality assessment and are able to apply that knowledge to various measures of student achievement” (p. 535). Based on Stiggins’s (1991) definition, the researchers from the language education community proposed the concept of LAL, noting that LAL is different from the assessment literacy in general education because the assessment of language knowledge and communicative competence has its own complexities (Jeong, 2013; Harding and Kremmel, 2016).

In recent decades, researchers have attempted to conceptualize LAL in different ways. Some conceptualized LAL as componential models (Brindley, 2001; Davies, 2008; Inbar-Lourie, 2008; Fulcher, 2012), while some regarded the development of LAL as a continuum (Pill and Harding, 2013). Brindley (2001) first identified five components of teachers’ LAL. The two core components consist of (i) an understanding of the social context of the assessment, and (ii) the ability to define and describe students’ language proficiency. The three optional components include the abilities to (iii) construct and evaluate language tests, (iv) develop assessment in the language curriculum, and (v) put assessment into practice. In light of the social turn in language testing (Roever and McNamara, 2006; Inbar-Lourie, 2008), Brindley (2001) emphasized the social role of the language assessment and put the understanding of “social, educational and political aspects of assessment” (p. 129) in the first place.

Adopting a social constructivist approach, Inbar-Lourie (2008) categorized Brindley’s (2001) five aforementioned components into three dimensions: the “what” (i.e., ii. Defining and describing students’ language proficiency), the “how” [i.e., (iii) Constructing and evaluating language tests, (iv) Assessment in the language curriculum, and (v) putting assessment into practice], and the “why” [i.e., (i) the social context of the assessment]. Knowing the “what” and operating the “how” can necessitate the comprehension of the social backgrounds and underlying reasons of the practices, the “why.” Davies (2008) also proposed that LAL consists of skills, knowledge and principles, which corresponds with Inbar-Lourie’s (2008) view noted above. In subsequent studies, skills, knowledge and principles are widely agreed as the core components of LAL (Giraldo, 2018; Deygers and Malone, 2019; Lee and Butler, 2020; Butler et al., 2021).

Based on his empirical findings, Fulcher (2012) expanded Davies’s (2008) conceptualization and proposed a three-tier hierarchical LAL model. Assessment practices, involving the knowledge, skills and abilities relevant to language assessments, are placed at the lowest level of the model. Assessment principles, which consist of assessment processes, principles and concepts, are placed in the intermediate level. The top layer of the model is the contexts, that is, the historical, social, political and philosophical contexts of language assessment. Echoing the previous LAL studies (Brindley, 2001; Inbar-Lourie, 2008), Fulcher’s (2012) model also underlined that language assessment should be understood “within a larger historical, social, political, and ethical framework” (p. 126). However, in contrast to the LAL components identified by Brindley (2001), which regarded the understanding of social context as a basic requirement, Fulcher (2012) considered it the highest requirement and not essential for every stakeholder group.

Prior conceptualizations had mostly viewed LAL as a dichotomy and considered people as either literate or illiterate. This issue was later addressed by Pill and Harding (2013), who regarded LAL as a continuum and identified five stages of LAL development: illiteracy, nominal literacy, functional literacy, procedural and conceptual literacy, and multidimensional literacy. This model provides a “literacy ladder” for assessment.

Integrating the componential view (Brindley, 2001; Davies, 2008; Inbar-Lourie, 2008; Fulcher, 2012) and the developmental view (Pill and Harding, 2013) of LAL, Taylor (2013) conceptualized LAL as eight dimensions and five stages. The eight dimensions include but are not limited to knowledge of theory, technical skills and sociocultural values. The five stages, consistent with Pill and Harding (2013), range from illiteracy to multidimensional literacy, with corresponding values from 0 to 4. For example, according to Taylor’s (2013) hypothesized LAL profile of classroom teachers, language pedagogy is the most important competency for classroom teachers. Technical skills, personal beliefs and attitudes, and local practices also constitute critical dimensions. Taylor’s model encourages people to consider LAL profiles in terms of stakeholder groups (Kremmel and Harding, 2020; Csépes, 2021), who require different LAL depending on their actual needs.

Along with the various dimensions and stages of LAL, the process of LAL development should be considered. Therefore, Yan and Fan (2021) proposed an apprenticeship-based, experience-mediated model. As indicated by this model, every stakeholder has a basic level of LAL influenced by their previous assessment experiences. In order to conduct appropriate assessment practices in a specific context, stakeholders should not only utilize their LAL knowledge base but also get familiar with local contexts and adapt their plans accordingly. These stakeholders’ assessment practices in local contexts in turn provide stakeholders with new assessment experiences, develop their LAL, and offer an opportunity to further reflect and evaluate their own assessment practices. Compared with previous conceptualizations, Yan and Fan (2021) paid greater attention to the interplay between LAL and various factors and identified the contextual and experiential factors. However, the real situation can be much more complex. Stakeholders’ cognitive traits and affective factors of language may also exert an influence during this process (Xu and Brown, 2016; Vogt et al., 2020).

Significant progress has been made in conceptualizing LAL, with the multidimensional and contextual dynamic nature of LAL recognized. However, some issues and challenges with the current LAL definitions and conceptualizations persist. First, the majority of existing LAL conceptualizations are proposed based on English-speaking contexts. However, because LAL is a highly contextualized concept, the LAL dimensions teachers need will vary from one context to another. In addition, further exploration is needed to conceptualize skill-specific LAL. Current LAL models are primarily concerned with general language assessment. However, assessing different language skills necessitates different knowledge and competence (Firoozi et al., 2019). For example, when developing listening tests, test developers should consider the phenomena associated with spoken language such as dialects, accents and regional variations, as well as colloquial language and slang (Wagner, 2013), which will not be considered when developing writing tests.

## Empirical Studies

### Language Teachers’ LAL

As one of the central stakeholder groups in the language assessment process, language teachers play an important role in language assessment and need to deal with various language assessment tasks. Recognizing the importance of language teachers’ LAL, the majority of relevant studies have focused on language teachers. Their LAL levels, as well as the approaches to increase those levels, have become two of the most discussed topics.

#### LAL Levels

Numerous studies have been done to investigate whether language teachers’ LAL levels are sufficient to fulfill their academic responsibilities (Cumming, 2001; Cheng et al., 2004; Alkharusi et al., 2011; Kiomrs et al., 2011; Vogt and Tsagari, 2014; Tsagari and Vogt, 2017; Xu and Brown, 2017; Homayounzadeh and Razmjoo, 2021). The most frequently used instruments are surveys, the majority of which are developed based on assessment literacy frameworks in general education. Interviews have also become prevalent in recent years. Prior studies have found that most language teachers had insufficient LAL. Some reported that teachers incorrectly understood language assessment (Kiomrs et al., 2011; Berry et al., 2017), did not acquire theoretical language assessment knowledge (Mede and Atay, 2017; Xu and Brown, 2017; Kim et al., 2020), designed language assessment intuitively (Sultana, 2019) or inappropriately interpreted students’ test results (Kim et al., 2020).

Aside from the investigations of general LAL levels, several studies have looked into teachers’ LAL in specific language skills, such as listening, reading, writing and speaking (Ho and Yan, 2021). For example, several studies have paid attention to teachers’ LAL in writing assessment (Crusan et al., 2016; Lam, 2019; Wang et al., 2020). Interestingly, the studies on teachers’ writing assessment literacy showed that most teachers displayed relatively adequate writing assessment literacy, though they still needed to improve in specific areas, such as designing rubrics (Crusan et al., 2016) and administering the assessment as learning (Lam, 2019). In addition, Shahzamani and Tahririan (2021) investigated Iranian Medical English for specific purposes (ESP) practitioners’ LAL in reading comprehension. The findings indicate that there is no significant difference in the way language teachers and content teachers assess students’ reading abilities.

This line of research reveals that in most cases, language teachers display insufficient LAL, and the findings could serve as a starting point for future studies on teachers’ professional development in language assessment. However, there are also some limitations. Firstly, most of the survey instruments used in these studies were not specifically designed to assess teachers’ LAL; rather, the items were applicable to assessment literacy in all fields. Therefore, the responses of these surveys may only reveal the general assessment literacy of language teachers. Furthermore, the current research relied mostly on self-reported data from surveys and interviews, while language teachers may be unable to precisely assess their own LAL levels and the results may be biased.

#### Factors Influencing LAL

Researchers have identified two major factors that influence teachers’ LAL: individual factors and contextual factors (Crusan et al., 2016). Several studies have investigated the influences of the individual factors on LAL (Crusan et al., 2016; Xu and Brown, 2017; Afshar and Ranjbar, 2021) and indicated that teachers’ linguistic backgrounds, years of teaching (Crusan et al., 2016), academic degrees, training experiences and fields of study (Afshar and Ranjbar, 2021) can significantly affect their LAL. Regarding contextual factors, the assessment cultures in different countries (Sultana, 2019; Tsagari, 2021), the educational landscapes and policies at the national level and the local level (Carless, 2012; Gu, 2014; Yan et al., 2018), school policies (Mansouri et al., 2021), institutional mandates (Yan et al., 2018) and the infrastructures provided by institutions (Firoozi et al., 2019) can influence teachers’ LAL in different ways.

The findings of this line of research have provided significant references for future research on how teachers’ LAL dimensions are affected by various factors. Up to now, most research in this field has been conducted in Asia, where an exam-oriented culture predominates, while other countries have received minimal attention. Furthermore, the cross-sectional design is often used despite the long-lasting influences of contextual and individual factors. Therefore, longitudinal studies are required in the future to determine the effects of various factors over time.

### Teachers’ LAL Development

Because the majority of language teachers do not have adequate LAL (Vogt and Tsagari, 2014; Lam, 2015; Crusan et al., 2016; Berry et al., 2017; Xu and Brown, 2017), figuring out how to enhance teachers’ LAL has become a primary concern. This section will examine the studies on language teachers’ assessment training needs, the effectiveness of existing assessment training courses and the use of self-reflection to improve LAL.

#### Language Assessment Training Needs

Designing an effective language assessment training program requires an understanding of what language teachers need (Ölmezer-Öztürk and Aydin, 2018; Gan and Lam, 2020). A number of relevant studies have been undertaken (Hasselgreen et al., 2004; Mendoza and Arandia, 2009; Jin, 2010; Fulcher, 2012; Vogt and Tsagari, 2014; Gan and Lam, 2020; Vogt et al., 2020; Zulaiha and Mulyono, 2020). For example, Hasselgreen et al. (2004) investigated teachers’ assessment training needs in Europe and the findings showed that teachers urgently needed knowledge about alternative assessments, such as portfolio, peer assessment and self-assessment.

Due to the highly contextualized nature of LAL, language teachers in different education systems require different types of assessment training (Vogt and Tsagari, 2014; Mede and Atay, 2017; Tsagari and Vogt, 2017; Xu and Brown, 2017). For example, according to Vogt and Tsagari’s research (Vogt and Tsagari, 2014; Tsagari and Vogt, 2017), foreign language (FL) teachers in Greece, Germany, and Cyprus had various expectations regarding LAL training. In Greece, FL teachers desired advanced training in classroom-based assessment activities because they faced high demands from the Ministry of Education. In contrast, German and Cypriot FL teachers had relatively moderate LAL training needs. German FL teachers placed an emphasis on reading and writing assessment because these were the primary focus of the German school leaving certificate (Abitur) test. In Cyprus, because FL teachers rarely developed exams on their own and usually modeled and applied existing large-scale international language exams, they lacked enthusiasm for advanced language assessment training.

Teachers of different educational stages also have different assessment training demands (Berry et al., 2017; Yan et al., 2018; Xie and Tan, 2019; Gan and Lam, 2020). For instance, in China, secondary language teachers reported that they preferred training on assessment practices instead of taking assessment knowledge courses (Yan et al., 2018; Lan and Fan, 2019), whereas college English instructors desired advanced training in assessment theories and concepts (Gan and Lam, 2020). The disparity is probably because English teachers in Chinese secondary schools and universities have varying daily tasks and career goals.

This line of research has illustrated the language assessment training requirements of teachers in various contexts. These findings are critical for future teacher education programs, since effective language assessment training programs cannot be offered without a thorough understanding of teachers’ actual needs. Currently, most of the relevant studies have surveyed or interviewed language teachers about their assessment training needs. However, language teachers did not always realize what they need or deemed everything presented to them to be necessary (Fulcher, 2012). Therefore, in future studies, viewpoints from other stakeholders who work closely with language teachers can also be adopted to contribute different angles.

#### Language Assessment Courses

A number of language assessment courses have been developed over the last few decades. Relevant studies have focused on the overall trend of language assessment courses (Bailey and Brown, 1996; Brown and Bailey, 2008) and the features of existing language assessment courses (e.g., Kremmel et al., 2018; Giraldo, 2021).

Bailey and Brown firstly conducted two similar studies to examine the characteristics of language assessment courses around the world and how they have changed over time (Bailey and Brown, 1996; Brown and Bailey, 2008). They found that the topics of language assessment courses remained stable during these years, expanding gradually rather than dramatically. In general, the topics of “classroom testing practices,” “testing in relationship to curriculum,” and “measuring the different skills” received the most coverage in language assessment courses.

The analysis of recent literature has also identified several features of language assessment courses. First, the vast majority of language assessment programs were designed for in-service language teachers (Nier et al., 2009; Baker and Riches, 2018; Giraldo, 2021), with fewer programs for pre-service teachers (Walters, 2010; Bolívar and Restrepo, 2020) and student teachers (O’Loughlin, 2006; Walters, 2010). However, pre-service teachers and student teachers should receive more language assessment training since they may be expected to conduct assessment tasks early in their careers. Second, the most common topics covered in these programs are evaluating and critiquing language assessments, designing language assessments, and writing items (Kremmel et al., 2018; Levi and Inbar-Lourie, 2020). The principles such as assessment fairness and ethics in language assessment are relatively neglected. Third, the majority of training courses were one semester in duration or shorter (e.g., Nier et al., 2009; Walters, 2010; Giraldo and Murcia, 2019; Levi and Inbar-Lourie, 2020), with fewer programs lasting several years (Kremmel et al., 2018). However, the long-term impact of short-term teacher training programs has been questioned in relevant research (e.g., Giraldo, 2021). Fourth, most training programs were face-to-face, with a few attempting to integrate the online resources and the on-site training (O’Loughlin, 2006; Nier et al., 2009). Many teachers, however, may be unable to attend on-site training courses due to a variety of factors such as severe workloads or geographical distances. Therefore, more flexible training modes should be proposed in the future. One example is teacher reflection and relevant research will be discussed in the next section.

#### Developing LAL Through Reflection

Reflection, an important approach for teachers’ professional development (Jamil and Hamre, 2018), allows teachers to stop and think about where they are and where they want to go (Farrell, 2012). In recent years, teacher reflection has gained scholarly attention and has been regarded as a compensation strategy for improving LAL (Babaii and Asadnia, 2019; Tian et al., 2021), because frequent reflection on assessment practice could help teachers recognize their own biases toward assessment and reconsider the incorporation of language assessment, teaching and learning (Scarino, 2013).

In a duoethnographic study by Tian et al. (2021), teacher researchers from three different countries attempted to investigate how reflection may affect teachers’ LAL. They found that reflecting on oneself and seeking opinions from others who were facing similar challenges but in different contexts could help them build confidence and gain the ability to handle assessment dilemmas. Reflection also enabled these teacher researchers in better understanding the connection between alternative assessment and teaching objectives. Another study by Babaii and Asadnia (2019) discovered that after reflecting on language assessment theories and practices, teachers became more autonomous and felt “more empowered to have their own agency in the language assessment process” (p. 758). Additionally, Yan and Fan (2021) suggested that self-reflection could help English as a foreign language teachers improve their understanding of language assessment and connect it to teaching and learning.

So far, research exploring how to improve teachers’ LAL through reflection has still been in its early stages. The current research has focused on teachers’ self-organized reflection activities, which did not rely much on external resources such as expert guidance or language assessment courses. Therefore, this type of reflection is more ideal for teachers who already possess a certain degree of LAL. To assist language teachers with limited LAL levels in developing their competencies, training programs that incorporate reflection sections alongside formal training courses will be more effective.

## Conclusion and Future Directions

While research on language teachers’ LAL has substantially expanded our knowledge of the field over the past decades, more efforts are required in the future. Several research directions for future studies are hereby suggested.

It is found that current LAL conceptualizations primarily focus on teachers’ competencies in assessing *general* language ability. Given the distinctiveness of different language abilities (i.e., listening, speaking, reading, and writing), future research should propose skill-specific LAL conceptualizations. In addition, most survey instruments employed in current LAL research are based on assessment literacy frameworks in general education. To more accurately assess teachers’ LAL, future research should develop LAL-specific survey tools that take into consideration the peculiarity of language assessment.

Moreover, whereas the factors that influence teachers’ LAL were extensively investigated in Asian countries, we still know very little about the factors affecting teachers’ LAL in other sociocultural contexts. As teachers from different contexts face varying assessment cultures, educational policies, students’ language levels, and so forth, more LAL investigations of teachers from other countries should be conducted.

Additionally, in current studies, many language teachers were unable to determine which language assessment skills or knowledge they need to enhance. Hence, the perspectives of other key stakeholders such as teacher educators (Bøhn and Tsagari, 2021) and university administrators also merit academic research attention. Researchers can also examine language teachers’ assessment processes, self-designed assignments, final papers, and how they use assessment results to identify their language assessment training needs.

Finally, the majority of the language teacher assessment training programs were shorter than one semester in duration. Although short-term training programs impart language assessment knowledge and skills, long-term training programs are needed as they are more effective at helping teachers apply what they have learned to the context in which they work. Flexible training methods such as online training courses, collaborative learning, and reflection are also worth exploring to ensure that as many language teachers as possible participate in LAL training.

## Author Contributions

FW conceived the paper and took the lead in writing the manuscript. BS revised critically for important intellectual content. All authors contributed to the article and approved the submitted version.

## Conflict of Interest

The authors declare that the research was conducted in the absence of any commercial or financial relationships that could be construed as a potential conflict of interest.

## Publisher’s Note

All claims expressed in this article are solely those of the authors and do not necessarily represent those of their affiliated organizations, or those of the publisher, the editors and the reviewers. Any product that may be evaluated in this article, or claim that may be made by its manufacturer, is not guaranteed or endorsed by the publisher.
